# Novel Pretreatment Autoantibodies Correlate with Enfortumab Vedotin–Related Dermatologic Events in Patients with Advanced Urothelial Cancer

**DOI:** 10.1158/2767-9764.CRC-25-0039

**Published:** 2025-09-18

**Authors:** Evangelia Vlachou, Burles A. Johnson, David J. McConkey, Noah M. Hahn, Yuezhou Jing, Stephanie Russell, Daniel Stairiker, Antony Rosen, Livia A. Casciola-Rosen, Jean Hoffman-Censits

**Affiliations:** 1 Department of Urology, The Johns Hopkins Greenberg Bladder Cancer Institute, Baltimore, Maryland.; 2 Department of Oncology, Johns Hopkins University Sidney Kimmel Comprehensive Cancer Center, Baltimore, Maryland.; 3 The James Buchanan Brady Urological Institute, Johns Hopkins University, Baltimore, Maryland.; 4 Sibley Memorial Hospital, John Hopkins Medicine, Washington, District of Columbia.; 5 Division of Rheumatology, Department of Medicine, Johns Hopkins University School of Medicine, Baltimore, Maryland.

## Abstract

Enfortumab vedotin (EV) plus pembrolizumab (P) is the first-line treatment for patients with advanced urothelial cancer (aUC). No biomarker for EV toxicity or response has been prospectively validated. EV-related dermatologic events (EVDE) are common and correlate with improved outcomes in retrospective studies. Immune checkpoint inhibitor–related toxicity also correlates with improved outcomes, and the presence of autoantibodies correlates with improved responses. We hypothesized that EV unmasks preexisting subclinical autoimmune responses leading to EVDEs in the setting of EV or EV/P. Our goal was to determine whether novel autoantibodies, if detected, correlate with EVDEs. We screened for novel autoantibodies using an immunoprecipitation-based approach in a pilot retrospective cohort (A) and a prospective longitudinal cohort (B) of patients with aUC who were treated with EV or EV/P and experienced EVDEs. Incidence of EVDEs, radiographic response, and progression-free and overall survival were compared between patients with and without autoantibodies. Two of six cohort A patients had pretreatment autoantibodies [Rho-associated coiled-coil containing protein kinase 2 (one patient) and target of Myb1-like 1 membrane trafficking protein (one patient)]. Pretreatment autoantibodies were also identified in 6/23 cohort B patients [mitochondrial complex 3 – M2 antigen (four patients), NMD3 ribosome export adapter (two patients), and carnitine palmitoyltransferase (one patient)]. All six (100%) cohort B antibody-positive patients developed EVDEs during treatment. To our knowledge, this is the first study correlating EVDEs with novel pretreatment autoantibodies in patients with mUC. Further studies are needed to confirm that they are biomarkers of EV-related skin toxicity and/or response.

**Significance:**

EV is approved alone and with pembrolizumab (EV/P) for aUC. EV-related dermatologic events (EVDEs) have been correlated with improved outcomes. We identified novel pre- and on-treatment autoantibodies in sera of patients with mUC treated with EV or EV/P which correlated with EVDEs.

## Introduction

For decades, metastatic urothelial cancer (mUC) of the bladder, urethra, ureters, and renal pelvis was associated with a poor median overall survival (OS) of 16 months with first-line platinum-based chemotherapy (1). In 2019, the FDA approved the antibody–drug conjugate (ADC) enfortumab vedotin (EV) for patients with mUC who experienced disease progression following platinum-based chemotherapy and immunotherapy, and in 2021 EV was also approved for mUC in cisplatin-ineligible patients with at least one prior line of therapy (2, 3). In late 2023, FDA approval was granted for EV in combination with the immune checkpoint inhibitor (ICI) pembrolizumab (P) for all patients with mUC (1). EV/P almost doubled median progression-free survival (PFS) and OS compared with platinum-based chemotherapy (1). EV is comprised of a human antibody targeting NECTIN4 that is linked to monomethyl auristatin E chemotherapy (4, 5). EV-related dermatologic events (EVDE) are very common toxicities and tend to appear in early cycles (3, 6, 7). EVDEs vary in severity and presentation and include maculopapular rash, blisters, dry skin, and hyperpigmentation, with rare life-threatening events (3, 6, 7).

Whereas NECTIN4 protein expression did not correlate with EV response in prospective trials, and no biomarker of toxicity or response has been prospectively validated (2, 8–11), almost all patients who had NECTIN4 amplification had an objective response to EV in a recent retrospective study (10). Our group previously reported that EVDEs correlated with improved radiographic response and survival in retrospective studies of patients treated with EV monotherapy, an association confirmed by other groups (12–14).

In many cancers, ICI-related immune toxicity correlates with better treatment response (15–17). Whereas ICI-mediated immune activation is not antigen-specific, ICI treatment resulted in autoantibody generation in patients, which associated with improved response (18). Moreover, higher numbers of autoantibodies in patients with anti-TIF1–positive dermatomyositis correlated with decreased cancer frequency (19). Thus, it has been proposed that diverse autoantibody responses are a marker of persistent immune editing that improves tumor control (20). However, whether ADC treatment stimulates unexpected phenotypes in patients with preexisting autoantibodies, or whether ADCs themselves stimulate autoantibody generation, is unexplored.

We hypothesized that EV precipitates EVDEs in the setting of EV or EV/P, either in patients with preexisting autoantibodies or in patients in whom treatment induces downstream autoantibody generation. To test this, we screened for the presence of novel autoantibodies in the sera of patients treated with EV and/or EV/P in a pilot retrospective cohort and a prospective longitudinal cohort. The five antibodies focused on in this article (see Table 2) were validated hits identified by proteomic analysis of on-bead digested immunoprecipitations (IP). Our goal was to determine whether novel autoantibodies correlated with EVDEs and/or improved treatment response.

## Materials and Methods

### Study design, participants, and data collection

This study was conducted in accordance with the Declaration of Helsinki and was approved by the Johns Hopkins Institutional Review Board. Written informed consent was obtained from patients in two separate cohorts. Pilot cohort A included patients with mUC treated with EV monotherapy at Johns Hopkins who had current or past EVDEs. One blood sample was collected from each patient after EVDE, and clinical data, including demographics, cancer stage and location, prior treatment, dosing, treatment response, and duration, were collected retrospectively by chart review.

Cohort B was a prospective longitudinal cohort. EV-naïve patients with mUC scheduled for EV or EV/P at Johns Hopkins were enrolled prior to C1D1. Baseline blood samples were collected before EV or EV/P therapy and before treatment for the first two cycles. Additional collections were performed at the time of EVDEs (Supplementary Fig. S1) if these occurred after cycle 2. Study data were collected prospectively and managed in the REDCap electronic data capture tool hosted at Johns Hopkins.

EVDEs were defined as any new or grade-escalated dermatologic event, including rash and pruritus after EV or EV/P initiation, not attributed to another cause. EVDE severity was assessed by the treating physicians according to the Common Terminology Criteria for Adverse Events v5.0.

### Antibody discovery and validation

The following approach was used for discovery. To test for the presence of autoantibodies, cohort A sera were used to perform exploratory IPs with ^35^S-methionine–radiolabeled MCF7 cell extracts as described (19). The immunoprecipitates were electrophoresed by SDS-PAGE and visualized by fluorography. The resulting profiles reflected the presence of autoantibodies against specific unidentified autoantigens in the sera. We therefore used this information to select a subset of sera for discovery of new autoantibodies. Discovery was performed using the following IP-based proteomic approach. IPs were done as described above using lysates made from unlabeled MCF7 cells. The amount of lysate and serum used per IP was scaled up fivefold relative to that used for the radiolabeled pilots. Subsequent processing steps, performed at the Johns Hopkins University Proteomics Core Facility, entailed on-bead digestions with trypsin/LysC and analysis of the resulting peptides by reverse-phase LC/MS as detailed (19). Putative hits from the discovery pipeline were subsequently rigorously validated as follows. Orthogonal validation of Rho-associated coiled-coil containing protein kinase 2 (ROCK2) was performed by IP and immunoblotting using an anti-ROCK2 mAb (Proteintech # 66633). To validate the target of Myb1-like 1 membrane trafficking protein (TOM1L1), DNA encoding full-length human TOM1L1 was used to generate ^35^S-methionine–labeled TOM1L1 by *in vitro* transcription and translation), and the *in vitro*–translated protein was used as the antigen source in IPs as described(19).

We subsequently used the same discovery strategy with sera from the first four patients evaluated in the prospective cohort. Based on exploratory IPs performed using radiolabeled cell extracts, sera from three patients were selected for proteomic analysis. This identified antibodies against NMD3 ribosome export adapter (NMD3), carnitine palmitoyltransferase (CPT1A), and the E2 component of pyruvate dehydrogenase complex (PDCE2)/dihydrolipoamide S-acetyltransferase (DLAT). These antibodies were confirmed in the index sera by *in vitro* transcription and translation and IP (NMD3), IP/immunoblotting (CPT1A and PDCE2/DLAT; rabbit antibodies used for blotting were Proteintech # 15184 and Novus NBP3-22153, respectively). The above assays were subsequently used to screen the cohorts for antibodies against ROCK2, TOM1L1, NMD3, and CPT1A. PDCE2/DLAT antibodies were evaluated using a commercially available ELISA kit for MIT3 antibodies [Quanta Lite M2 EP (MIT3) ELISA, INOVA-WERFEN, cat. # 704540]. This assay was chosen because of the nine types of mitochondrial antigens (M1–M9), the major autoantibodies in sera from patients with primary biliary cirrhosis are against the M2 antigen. Per information provided by the manufacturer, “MIT3” is a triple-expression hybrid clone. It expresses the immunodominant epitopes of three prominent components of the M2 antigen. These components are PDCE2/DLAT, BCOADCE2, and OGDC-E2. This ELISA is therefore widely used to read out PDCE2/DLAT antibodies. The data generated in this study are available upon request from the corresponding author.

### Statistical analysis

Summary statistics were used to summarize baseline characteristics in both cohorts. Response rate [RR = complete response (CR) + partial response (PR)] and disease control rate (DCR = CR + PR + stable disease) were calculated based on physician-assessed RECIST v1.1 for radiographic response and compared between patients with and without EVDEs and autoantibodies using two-tailed Fisher exact test.

Log rank test and Cox proportional HRs were used to compare PFS and OS between patients with and without EVDEs. PFS was defined as time in months from EV initiation until radiographic progression or death. EVDEs were assessed as a time-dependent variable to account for immortal time bias in the survival analysis. None of the patients in our cohort had clinical response without radiographic progression. Three patients died without radiographic progression and were included in our analysis. Patients who did not progress at cutoff were censored at the time of last scan without disease progression. OS was defined as time in months from EV initiation until death due to any cause, and patients who were still alive at cutoff were censored at the date of last follow-up.

Statistical analysis was performed using R v4.3.3 and SAS v9.4. Statistical significance was defined as *P* < 0.05.

### Data availability

The data generated in this study are available upon request from the corresponding author.

## Results

### Baseline characteristics

Between February and March of 2023, six patients with locally advanced or metastatic urothelial cancer (la/mUC) treated with EV monotherapy who experienced EVDEs were included in the pilot cohort A. Baseline characteristics are summarized in Supplementary Table S1.

From June 2023 until July 2024, 25 patients were prospectively assessed for eligibility for cohort B, and 23 were eligible for analysis at study cutoff (November 2024; Supplementary Fig. S2). Nineteen (82.6%) patients received EV/P, and 4 (17.4%) received EV monotherapy (2/4 received prior pembrolizumab, 8 days and 2 months, respectively, before initiating EV). The median age for the cohort was 70.8 years (IQR, 64–76). Sixteen (69.6%) patients were male, and 18 (78.3%) had visceral metastases at baseline. Nineteen (82.6%) patients experienced any-grade EVDEs, and 11 (47.8%) patients required EVDE-directed intervention, including dose modifications, treatment breaks, systemic and topical antihistamines, and topical or systemic steroids. Baseline characteristics were similar between patients with and without EVDEs (Table 1) and autoantibodies (Supplementary Table S2).

**Table 1.** Baseline characteristics of patients with vs. without EVDEs (cohort B).

​	No EVDEs *N* = 4	EVDEs *N* = 19	*P* value^a^
Age, median (IQR)	66.5 (64.3–69.8)	73.2 (65.4–76.4)	0.37
Gender, *n* (%)			0.56
Female	2 (50.0)	5 (26.3)	​
Male	2 (50.0)	14 (73.7)	​
Race, *n* (%)			0.23
White	3 (75.0)	16 (84.2)	​
Black	0 (0.0)	3 (15.8)	​
Asian	1 (25.0)	0 (0.0)	​
Tumor location, *n* (%)	​		0.39
UTUC	0 (0.0)	7 (36.8)	​
Bladder, lower tract	4 (100.0)	11 (57.9)	​
Both	0 (0.0)	1 (5.3)	​
Visceral disease, *n* (%)	4 (100.0)	14 (73.7)	0.54
Lung	3 (75.0)	9 (47.4)	0.59
Liver	1 (25.0)	6 (31.6)	1.00
Bone	2 (50.0)	4 (21.1)	0.27
Peritoneum	0 (0.0)	1 (5.3)	1.0
Brain	1 (25.0)	0 (0.0)	0.17
ECOG PS, *n* (%)	​	​	0.088
0	2 (50.0)	13 (68.4)	​
1–2	2 (50.0)	6 (31.6)	​
EV line, *n* (%)			1.0
First-line	3 (75.0)	10 (52.6)	​
Second-line	1 (25.0)	6 (31.6)	​
Third-line	0 (0.0)	3 (15.8)	​
EV initiation at full dose, *n* (%)^b^	4 (100.0)	16 (84.2)	1.0
Regimen, *n* (%)	​	​	1.0
EV alone	1 (25.0)	3 (15.8)	​
EV + P	3 (75.0)	16 (84.2)	​

Abbreviations: ECOG PS, Eastern Cooperative Oncology Group Performance Status; UTUC, upper tract urothelial cancer.

a Based on Fisher exact test for categorical variables and Wilcoxon test for continuous variables.

b Full dose is 1.25 mg/kg capped at 125 mg.

### EVDEs and autoantibodies

**Figure 1.**
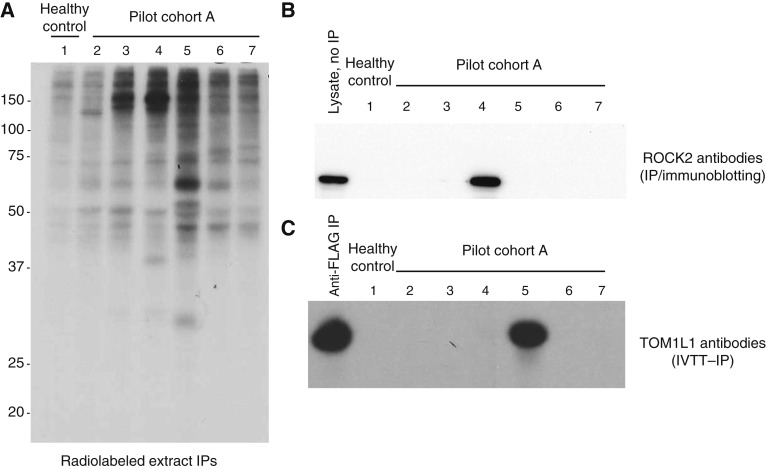
IPs from radiolabeled cell extracts using the pilot cohort sera (**A**), and validation of ROCK2 (**B**) and TOM1L1 (**C**) antibodies in the index sera. **A,** IPs were performed using ^35^S-methionine–labeled MCF7 cell extracts and patient sera from the pilot cohort (sera #2–7) or a healthy control serum (#1). The IPs were electrophoresed by 10% SDS-PAGE, and immunoprecipitated proteins were visualized by autoradradiography. Migration of molecular weight standards are marked on the left. **B,** Antibodies against ROCK2 in index serum #4 from the pilot cohort were confirmed by IP from MCF7 lysates, followed by blotting with an anti-ROCK2 mAb. As a positive reference, the input lysate (lane denoted “lysate, no IP”) was included. **C,** The presence of TOM1L1 antibodies in index serum #5 from the pilot cohort was confirmed by IP using ^35^S-methionine–labeled TOM1L1 generated by *in vitro* transcription and translation (IVTT). As a positive control, an anti-FLAG IP was performed (the IVTT protein is FLAG-tagged). The data in (**B**) and (**C**) confirm the presence of ROCK2 and TOM1L1 antibodies in sera #4 and #5, respectively.

We tested for the presence of autoantibodies by IP from radiolabeled cell extracts using the six sera in the pilot cohort A. Several proteins were immunoprecipitated, reflecting the presence of autoantibodies in these sera (Fig. 1A). We then selected sera that immunoprecipitated the most prominent bands (Fig. 1A, sera #4 and 5) for antibody discovery (see “Materials and Methods” section). This identified antibodies against ROCK2 (in #4) and TOM1L1 (in #5), which we subsequently validated (Supplementary Table S2). The pilot cohort was tested using the two assays set up for validation; these antibodies were uniquely found in the index cases.

The pilot data suggested that the presence of autoantibodies in patients with this clinical phenotype warranted further investigation in a prospective cohort. However, these samples were taken after EVDEs. As there was no baseline or serial collection, it was unclear whether these autoantibodies were preexisting or dynamic, e.g., detectable only at certain points in the treatment time course. To address these issues, we used the approach described above for autoantigen discovery in longitudinal cohort B sera. For this, we screened baseline and on-treatment sera from the first four patients in the cohort by IP from radiolabeled cell extracts. These data were used to select a subset of these sera for autoantibody discovery. Autoantibodies against NMD3, CPT1A, and PDCE2/DLAT were identified with this proteomic approach and subsequently validated in the index sera. When the prospective cohort was assayed for ROCK2, TOM1L1, and these three antibodies, 6/23 (26.1%) patients tested positive (Supplementary Table S3). One patient (EV-17) was positive for two different autoantibodies (Supplementary Table S4). In one patient (EV-8), the anti-MIT3 antibody assay was negative/borderline for the first four bleeds and subsequently became positive. In the other three anti-MIT3–positive patients, antibody levels remained fairly constant over time. Anti-NMD3 antibodies were present at similar levels in all sera from patient EV-4 but decreased over time in patient EV-17. In patient EV-1, anti-CPT1A antibody levels increased after visit 1 and remained constant thereafter. For one patient (EV-1), the baseline sample was not available, but autoantibodies were identified in all subsequent samples tested. Two of six (33.3%) antibody-positive patients were initially treated with EV monotherapy, and 4/6 (66.7%) with EV/P. All six (100%) patients with autoantibodies present at baseline developed EVDEs during treatment (Table 2). When pilot cohort A was tested for autoantibodies against NMD3, CPT1A, and MIT3, none were detected.

**Table 2.** Autoantibody presence at baseline in patients in cohorts A (*n* = 6) and B (*n* = 23) with vs. without EVDEs and in patients with EVDEs requiring intervention.

*N*(%)	No EVDEs	EVDEs	EVDEs requiring intervention^a^
Cohort A^b^	NA	*N* = 6	*N* = 4
Autoantibodies	NA	2 (33.3)	1 (25)
ROCK2	NA	1 (16.7)	0 (0.0)
TOM1L1	NA	1 (16.7)	1 (25)
Cohort B^c^	*N* = 4	*N* = 19	*N*= 11
Autoantibodies	0 (0.0)	6 (31.6)	2 (18.2)
NMD3	0 (0.0)	2 (10.5)	1 (9.1)
MIT3	0 (0.0)	4 (21.1)	2 (18.2)
CPT1A	0 (0.0)	1 (5.3)	1 (5.3)

a Interventions included topical and/or systemic antihistamines, topical and/or systemic steroids, treatment breaks, and dose modifications due to EVDEs.

b All patients in cohort A tested negative for NMD3, MIT3, and CPT1A.

c All patients in cohort B tested negative for ROCK2 and TOM1L1.

Of note, all patients in the prospective cohort (cohort B) that had a detectable autoantibody and an available baseline sample had the same autoantibody at baseline. The only patient without a baseline sample had an autoantibody detectable at C1D8. Moreover, these baseline autoantibodies were detectable at all subsequent laboratory draws.

### EVDEs and cancer outcomes

Cohort A only included patients with EVDEs. The RR and DCR were 5/6 (83.3%), including 1 (16.7%) CR and 4 (66.7%) PR (Supplementary Table S1).

In the entire cohort B, the RR was 17/23 (73.9%) and DCR was 21/23 (91.3%). The RR was numerically higher among patients who experienced EVDEs compared with patients who did not (78.9% vs. 50%, *P* = 0.27; Table 3). The DCR was significantly higher among the EVDE group (100% vs. 50%, *P* = 0.02). Radiographic response for patients with and without autoantibodies is presented in Supplementary Table S5.

**Table 3.** Radiographic response in cohort B patients with vs. without EVDEs (*n* = 23).

*N* (%)	No EVDEs No. 4	EVDEs No. 19	*P* value^a^
RR (CR + PR)	2/4 (50%)	15/19 (78.9)	0.2705
CR	1 (25)	3 (15.8)	​
PR	1 (25)	12 (63.2)	​
DCR (CR + PR + SD)	2/4 (50%)	19/19 (100%)	0.02372
SD	0 (0)	4 (21.1)	​
PD	2 (50)	0 (0)	​

Abbreviations: PD, progression of disease; SD, stable disease.

a Based on Fisher exact test.

For cohort B, the median PFS was 6.5 months [95% confidence interval (95% CI), 2.8–8.4] and the median OS was 9.4 months (95% CI, 5.9–12.4). PFS was significantly longer for patients with EVDEs compared with patients without (median PFS 6.5 vs. 2.8; HR = 0.16; 95% CI, 0.04, 0.7; *P* = 0.02). The OS was 9.4 for the EVDE group and NR for the no EVDE group with relatively short follow-up (HR = 0.59; 95% CI, 0.06–5.69; *P* = 0.6).

## Discussion

In serum samples of a pilot cohort of patients with EVDEs, we identified novel autoantibodies, and subsequently in an expanded prospective longitudinal cohort, we identified autoantibodies pre- and on-treatment. To our knowledge, this is the first study to report the presence of autoantibodies correlating with EVDEs. It is noteworthy that all target antigens (ROCK2, TOM1L1, NMD3, CPT1A, and MIT3) are expressed in normal skin according to the Human Protein Atlas (21), consistent with a possible relationship between autoantibodies and skin toxicity. Importantly, these antibodies were present before treatment initiation, although additional studies are needed to determine whether autoantibody presence, titer, and/or specificity predict EVDEs. Furthermore, all antibody-positive patients had at least some initial response to treatment, whereas 2/2 patients with primary progressive disease as best response had no EVDEs and no detectable autoantibodies. As autoantibody-driven clonal T-cell expansion may contribute to EVDEs, experiments to identify T-cell clones (if any) that respond to these unique antigens may assist in further understanding this mechanism.

We hypothesize that an autoantibody-producing B cell–driven process, modulated by EV, increases susceptibility to EVDEs. Autoantibody production may correlate with enhanced immune system response against antigens (self and by extension tumor) present before treatment initiation. Tumor destruction caused by EV theoretically increases the tumor antigen load, while causing inflammation of specific tissues like the skin, leading to EVDEs. Chemotherapy increased peripheral B-cell and other antigen-presenting cell activation programs in patients with mUC (22). Moreover, chemotherapy expanded antitumor B cells expressing ICOS ligand in patients with breast cancer (23), and ICOS ligand(+) B cells correlated with disease severity in rheumatoid arthritis (24). Thus, a B-cell population has been associated with antitumor immunity and autoimmune disease severity, similar to our autoantibody-expressing cohort in which we found disease response and EVDEs. As mUC is an aggressive disease that currently lacks validated clinical or molecular biomarkers predictive of EV or EV/P response, future analyses into the relationship between autoantibodies, clinical response, and skin toxicity has the potential to inform the development of clinically significant biomarkers.

The presence of preexisting autoantibodies challenges the hypothesis that EVDEs are solely because of off-tumor effects, for example resulting from NECTIN expression on the skin (18, 25, 26). Although others have demonstrated that autoantibodies appeared after ICI treatment, we did not detect this in our prospective cohort of patients who were treated with EV/P. This may have been because of the small sample size in our cohort, as autoantibody detection after ICI treatment only occurred in about 19% of patients (18). Alternatively, mechanisms of ICI-mediated adverse events and EVDEs may be different, suggestive of independent mechanisms of action and consistent with the nonsynergistic clinical response observed in combination trials of ICI with other agents (27–30).

Study limitations include the single-center study with lack of external validation, small sample size and limited power for comparisons, short follow-up, and univariable analysis. Our discovery strategy would not capture all known autoantibodies present in a patient’s serum, and we cannot definitively differentiate EVDEs from events that could be attributed to pembrolizumab among patients treated with EV/P. This small hypothesis-generating study is the first to report that EVDEs correlate with preexisting autoantibodies. Larger controlled studies, and those that investigate the immunologic effects of the individual components of the EV/P regimen, are needed to fully evaluate the landscape of immune responses in these patients, their timing, and their clinical associations.

### Conclusion

Our study correlated EVDEs with novel pre- and on-treatment autoantibodies in retrospective and prospective la/mUC cohorts. Further studies are needed to expand the pool of discovery antibodies in this clinical spectrum and confirm whether autoantibodies are biomarkers of toxicity in patients with la/mUC treated with EV and/or EV/P.

## Supplementary Material

Supplementary Figure 1 Figure 1

Supplementary Table 1 Table 1

Supplementary Figure 2 Figure 2

Supplementary Table 2 Table 2

Supplementary Table 3 Table 3

Supplementary Table 4 Table 4

Supplementary Table 5 Table 5
