# Supplementary material for: Novel Pretreatment Autoantibodies Correlate with Enfortumab Vedotin–Related Dermatologic Events in Patients with Advanced Urothelial Cancer
Source: Cancer Res Commun. 2025 Sep 18;5(9):1674–80. doi: 10.1158/2767-9764.CRC-25-0039 (PMC12444012; doi:10.1158/2767-9764.CRC-25-0039)
Supplement: Supplementary Figure 1 — Figure 1 [file crc-25-0039_supplementary_figure_1_suppsf1.docx]

**Supplementary Figure 1.** Summary of longitudinal blood collections in Cohort B
