# Supplementary material for: Novel Pretreatment Autoantibodies Correlate with Enfortumab Vedotin–Related Dermatologic Events in Patients with Advanced Urothelial Cancer
Source: Cancer Res Commun. 2025 Sep 18;5(9):1674–80. doi: 10.1158/2767-9764.CRC-25-0039 (PMC12444012; doi:10.1158/2767-9764.CRC-25-0039)
Supplement: Supplementary Table 1 — Table 1 [file crc-25-0039_supplementary_table_1_suppst1.docx]

| Supplementary Table 1. Demographics, EVDE and treatment response in patients with and without autoantibodies in cohort A (n=6) | | | | | | | | | | | |
| --- | --- | --- | --- | --- | --- | --- | --- | --- | --- | --- | --- |
| Sample ^a^ | **Age** | **Gender** | **Race** | **EV Line** | **EVDE cycle** | **EVDE grade** | **Onset of EVDE to blood collection (months)** | **Best EV response ^b^** | **Systemic Steroids** | **Prior ICI** | **Auto- Abs** |
| 2 | 63 | M | White | 3 | 1 | 1 | 0.7 | PD | No | Yes | - |
| 3 | 71 | M | White | 3 | 1 | 3 | 2.6 | PR | Yes (for EVDEs) | Yes | - |
| 4 | 86 | M | Black or African American | 3 | 2 | 1 | 0.2 | PR | No | Yes | Anti-ROCK2 |
| 5 | 70 | F | Black or African American | 3 | 2 | 1 | 5.4 | PR | No | Yes | Anti-TOM1L1 |
| 6 | 38 | M | Hispanic or Latino | 3 | 2 | 3 | 1.6 | PR | Yes (for EVDEs) | Yes | - |
| 7 | 62 | M | White | 3 | 1 | 2 | 9.1 | CR | No | Yes | - |
| ^a^ Sample 1 was a healthy control; ^b^ Physician assessed radiographic response  C1D1: cycle 1 day 1; EV: enfortumab vedotin; EVDE: enfortumab-related dermatologic events; ICI: immune checkpoint inhibitor; Abs: antibodies; M: male; F: female; PD: progression of disease; PR: partial response; CR complete response | | | | | | | | | | | |
