# Supplementary material for: Novel Pretreatment Autoantibodies Correlate with Enfortumab Vedotin–Related Dermatologic Events in Patients with Advanced Urothelial Cancer
Source: Cancer Res Commun. 2025 Sep 18;5(9):1674–80. doi: 10.1158/2767-9764.CRC-25-0039 (PMC12444012; doi:10.1158/2767-9764.CRC-25-0039)
Supplement: Supplementary Table 2 — Table 2 [file crc-25-0039_supplementary_table_2_suppst2.docx]

| Supplementary Table 2. Baseline characteristics of patients with vs without autoantibodies (cohort B) | | | |
| --- | --- | --- | --- |
|  | **No AutoAb**  **N=17** | **AutoAb**  **N=6** | **P-value ^b^** |
| Age, median (IQR) | 73.2 (64.3 - 76.1) | 68.2 (64.5 - 74.5) | 0.52 |
| Gender, n(%) | | | 0.62 |
| Female | 6 (35.3) | 1 (16.7) |  |
| Male | 11 (64.7) | 5 (83.3) |  |
| Race, n(%) | | | 1 |
| White | 14 (82.4) | 5 (83.3) |  |
| Black | 2 (11.8) | 1 (16.7) |  |
| Asian | 1 (5.9) | 0 (0.0) |  |
| Tumor location, n(%) |  | | 0.51 |
| UTUC | 4 (23.5) | 3 (50.0) |  |
| Bladder - lower tract | 12 (70.6) | 3 (50.0) |  |
| Both | 1 (5.9) | 0 (0.0) |  |
| Visceral disease, n(%) | 13 (76.5) | 5 (83.3) | 1 |
| Lung | 9 (52.9) | 3 (50.0) | 1 |
| Liver | 4 (23.5) | 3 (50.0) | 0.32 |
| Bone | 4 (23.5) | 2 (33.3) | 0.63 |
| Peritoneum | 0 (0.0) | 1 (16.7) | 0.26 |
| Brain | 1 (5.9) | 0 (0.0) | 1 |
| ECOG PS, n(%) |  |  | 1 |
| 0 | 11 (64.7) | 4 (66.7) |  |
| 1-2 | 6 (35.3) | 2 (33.3) |  |
| EV line, n(%) | | | 0.19 |
| 1st Line | 11 (64.7) | 2 (33.3) |  |
| 2nd Line | 5 (29.4) | 2 (33.3) |  |
| 3rd Line | 1 (5.9) | 2 (33.3) |  |
| EV initiation at full dose, n(%) ^a^ | 13 (76.5) | 2 (33.3) | 0.5304 |
| ^a^ full does is 1.25 mg/kg capped at 125 mg; ^b^ Based on Fisher’s exact test for categorical variables and Wilcoxon test for continuous variables  EVDE: enfortumab-related dermatologic events; UTUC: upper tract urothelial cancer; P: pembrolizumab; ECOG PD: Eastern Cooperative Oncology Group Performance Status | | | |
