# Supplementary material for: Novel Pretreatment Autoantibodies Correlate with Enfortumab Vedotin–Related Dermatologic Events in Patients with Advanced Urothelial Cancer
Source: Cancer Res Commun. 2025 Sep 18;5(9):1674–80. doi: 10.1158/2767-9764.CRC-25-0039 (PMC12444012; doi:10.1158/2767-9764.CRC-25-0039)
Supplement: Supplementary Table 3 — Table 3 [file crc-25-0039_supplementary_table_3_suppst3.docx]

| Supplementary Table 3. Autoantibody targets identified in cohorts A (n=6) and B (n=23) | | | |
| --- | --- | --- | --- |
| Target | **Full Name** | | **Number of positive patients** |
| Cohort A | | | |
| ROCK2 | | Rho associated coiled-coil containing protein kinase 2 | 1 |
| TOM1L1 | | target of myb1 like 1 membrane trafficking protein | 1 |
| Cohort B | | | |
| MIT3 (M2 antigen)^b^ | | Mitochondrial complex 3 | 4 |
| NMD3 | | NMD3 ribosome export adaptor | 2 |
| CPT1A | | Carnitine palmitoyltransferase 1A | 1 |
