# Supplementary material for: Novel Pretreatment Autoantibodies Correlate with Enfortumab Vedotin–Related Dermatologic Events in Patients with Advanced Urothelial Cancer
Source: Cancer Res Commun. 2025 Sep 18;5(9):1674–80. doi: 10.1158/2767-9764.CRC-25-0039 (PMC12444012; doi:10.1158/2767-9764.CRC-25-0039)
Supplement: Supplementary Table 4 — Table 4 [file crc-25-0039_supplementary_table_4_suppst4.docx]

| Supplementary Table 4. Autoantibody positivity in cohort B | | | | | | |
| --- | --- | --- | --- | --- | --- | --- |
| Sample | **Regimen** | **Prior ICI** | **Timepoint** | **NMD3 (IVTTIP)** | **MIT3 ELISA**  **(3 proteins_ DBT/DLAT/)** | **CPT1A (Ipblot)** |
| EV-1 | EV/P | No | C1D8 | - | - | **+** |
|  |  |  | C2D1 | - | - | **+** |
|  |  |  | C2D8 | - | - | **+** |
| EV-4 | EV | Yes | Baseline | **+** | - | - |
|  |  |  | C1D8 | **+** | - | - |
|  |  |  | C1D15 | **+** | - | - |
|  |  |  | C2D1 | **+** | - | - |
|  |  |  | C2D8 | **+** | - | - |
|  |  |  | C2D15 | **+** | - | - |
| EV-8 | EV | No | Baseline | - | **+** | - |
|  |  |  | C1D8 | - | **+** | - |
|  |  |  | Treatment break due to EVDE | - | **+** | - |
|  |  |  | Treatment break due to EVDE | - | **+** | - |
|  |  |  | C2D1 | - | **+** | - |
|  |  |  | Treatment break due to mucositis | - | **+** | - |
|  |  |  | Treatment break due to mucositis | - | **+** | - |
| EV-13 | EV/P | No | Baseline | - | **+** | - |
|  |  |  | C1D8 | - | **+** | - |
|  |  |  | C2D1 | - | **+** | - |
|  |  |  | C2D8 | - | **+** | - |
| EV - 17 | EV/P | No | Baseline | **+** | **+** | - |
|  |  |  | C1D8 | **+** | **+** | - |
|  |  |  | Treatment break due to EVDE | **+** | **+** | - |
|  |  |  | Treatment break due to hypertension | **+** | **+** | - |
|  |  |  | C2D1 | **+** | **+** | - |
| EV-18 | EV+P | No | Baseline | - | **+** | - |
|  |  |  | C1D8 | - | **+** | - |
|  |  |  | Treatment break due to neuropathy | - | **+** | - |
|  |  |  | C2D1 | - | **+** | - |
|  |  |  | C2D8 | - | **+** | - |
|  |  |  | C2D15 | - | **+** | - |
|  |  |  |  |  |  |  |
