# Supplementary material for: Novel Pretreatment Autoantibodies Correlate with Enfortumab Vedotin–Related Dermatologic Events in Patients with Advanced Urothelial Cancer
Source: Cancer Res Commun. 2025 Sep 18;5(9):1674–80. doi: 10.1158/2767-9764.CRC-25-0039 (PMC12444012; doi:10.1158/2767-9764.CRC-25-0039)
Supplement: Supplementary Table 5 — Table 5 [file crc-25-0039_supplementary_table_5_suppst5.docx]

| Supplementary Table 5. Radiographic response in cohort B patients with vs without autoantibodies (n=23) | | | |
| --- | --- | --- | --- |
| N (%) | **Ab - N=17** | **Ab + N= 6** | **P-value*** |
| RR (CR+PR) | 13/17 (76.5%) | 4/6 (66.7%) | 0.6322 |
| CR | 4 | 0 |  |
| PR | 9 | 4 |  |
| DCR (CR+PR+SD) | 15/17 (88.2%) | 6/6 (100%) | 1 |
| SD | 2 | 2 |  |
| PD | 2 | 0 |  |
| ^a^ Based on Fisher’s exact test  Ab: antibody; RR: response rate; CR: complete response; PR: partial response; DCR: disease control rate; SD: stable disease; PD: progression of disease | | | |
